# From bench to babies – drug development for male subfertility

**DOI:** 10.1530/RAF-24-0022

**Published:** 2024-10-04

**Authors:** Shen Chuen Khaw, Sarah Martins da Silva

**Affiliations:** 1 Reproductive Medicine Research Group, School of Medicine, Ninewells Hospital and Medical School, University of Dundee, Dundee, UK; 2 Assisted Conception Unit, Ninewells Hospital, Dundee, UK

**Keywords:** male infertility, sperm, drug development, drug discovery, high throughput phenotypic screening

## Abstract

Infertility is estimated to affect more than 50 million couples around the world, with the male factor accounting for half of these cases, yet there is a notable absence of effective treatment options for men, other than in-vitro fertilisation (IVF) or intracytoplasmic sperm injection (ICSI). This review considers unlicensed and empirical treatments used for male subfertility, including hormonal therapy, phosphodiesterase inhibitors, and antioxidants. Compounds generally demonstrate variable improvements in sperm function, but benefits for fertility are less clear. There is a pressing need for effective treatment options for subfertile men; however, our knowledge of sperm function is limited, restricting the identification of precise treatment targets. The traditional drug discovery pathway is also notorious for its extensive resource and time requirements, often extending over decades and demanding significant financial investment. Unfortunately, a substantial number of potential therapies fail before reaching the marketplace. Furthermore, reliance on mammalian models is not possible in the drug development process for male subfertility, due to significant variability between animals and man. We review recent breakthroughs and highlight novel methods aimed at improving the effectiveness and efficiency of drug discovery for male subfertility. High-throughput screening, combinatorial chemistry, and the repurposing of established medications have great potential. These strategies offer the promise of accelerating the pace of drug development, curbing the extensive demand for resources, and, in the case of drug repurposing, diminishing the demand for comprehensive pharmacokinetic and pharmacodynamic studies. As these innovative approaches are adopted, the feasibility of addressing male subfertility through scientific advancements appears to be increasingly attainable.

**Lay summary:**

Globally, millions of men suffer from infertility, but with very few exceptions, there is no effective treatment or cure other than complex fertility treatments such as *in vitro* fertilisation. This review article considers various approaches to treat men, or sperm, which have been largely unsuccessful to date. A significant issue is that we lack a detailed understanding of how sperm work and why treatments do not work. Another issue is that drug discovery is slow and costly because many potential treatments fail early on. However, we discuss recent technical and technological advancements that offer hope, such as high-throughput and phenotypic screening, which allow for the rapid testing of thousands of chemicals to observe their effects on sperm, and combinatorial chemistry, which involves generating large numbers of compounds to find those with beneficial properties. Finding relevant new uses for prescription drugs could also significantly speed up the drug discovery process and result in much-needed new treatments for male infertility.

## Introduction

The World Health Organization estimates that one in six couples encounter difficulty in achieving pregnancy within 12 months of regular unprotected sexual intercourse. Infertility impacts around 50 million couples, affecting the lives of approximately 190 million individuals globally (Agarwal *et al.* 2019, The Lancet Global Health 2022). Male factor infertility is a significant contributor to overall cases of infertility, affecting up to 50% of couples who struggle to conceive. Moreover, in around half of the cases of male factor infertility, no specific aetiology can be found despite carrying out detailed clinical examinations and investigations (Cairo Consensus Workshop Group 2019).

The current management of male infertility predominantly revolves around *in vitro* fertilisation (IVF) with intracytoplasmic sperm injection (ICSI). However, treatment is exclusive to couples who possess the financial means to cover the substantial costs or those residing in countries where government healthcare funding facilitates access to assisted reproduction technology (ART) programmes. A large disparity is thus seen between individuals from affluent countries and low- and middle-income countries (LMIC) who encounter substantial barriers in obtaining fertility treatment (The Lancet Global Health 2022). It is imperative that more affordable therapies are developed to reduce global inequalities in accessing treatment. Moreover, infertile men express a desire for alternative treatment options (Martins da Silva 2019).

This article delves into the challenges associated with the development of pharmaceutical interventions aimed at improving male fertility. We describe the multifaceted obstacles faced in this field and explore potential strategies and innovative approaches that could pave the way to overcome the challenges in addressing idiopathic male infertility (IMI). By examining the complexities involved, we aim to contribute valuable insights to the ongoing discourse surrounding male reproductive health and offer guidance for future research endeavours in this critical domain.

## Navigating complexities in drug development for enhancing male fertility

### Searching in the dark – what are we actually looking for?

Clinicians often grapple with a fundamental question: ‘What constitutes a good semen sample?’ Traditionally, the answer has been sought in diagnostic semen analysis. However, it is well established that manual semen analysis is inherently subjective, carries inaccuracies, and can be prone to error. Even seemingly mundane aspects like sample collection methods, adherence to recommended abstinence periods, and timely delivery to the laboratory can introduce biases, skewing evaluation results (Barratt *et al.* 2011, Cairo Consensus Workshop Group 2019). Importantly, whilst semen analysis results offer some guidance regarding male fertility potential, they fall short of thoroughly assessing the true function and fertilisation potential of spermatozoa. Approximately 10–15% of infertile men present semen parameters within the normal reference range, yet their infertility remains unexplained. A significant limitation is that fertility requires effective reproductive function in both males and females. Current trials often focus on surrogate markers of male fertility potential, such as sperm count, motility, and morphology. However, factors critical to successful fertilisation such as DNA integrity, capacitation, acrosomal reaction, hyperactivation, and cell signalling currently remain unexplored by traditional semen analysis (Lamb & Marinaro 2023).

In light of these challenges, defining what constitutes a ‘good’ semen sample becomes a pressing necessity. Recognising the shortcomings of traditional semen analysis, the scientific community increasingly calls for a more comprehensive approach in developing tests to evaluate spermatozoa function (Barratt *et al.* 2021). Targeted drug interventions to enhance spermatozoa function and factors influencing fertilisation remain elusive. Until this changes, drug development for male infertility remains focussed on improving spermatozoa concentration and/or motility.

### Nature’s master of variation

Developing drugs for male infertility faces significant challenges due to considerable variability in spermatozoa characteristics, both between individuals and between ejaculates from the same person. This heterogeneity is evident under microscopic examination, revealing distinct motions and morphologies among spermatozoa samples. One of the problems with using semen analysis as the primary outcome in a drug trial is the phenomenon known as ‘regression to the mean’. This statistical effect arises due to random biological fluctuation, yet can influence baseline measurements and the interpretation of trial results, potentially giving an illusion of a therapeutic benefit where, in point of fact, none exists.

Moreover, computer-assisted semen analysis (CASA) further classifies ejaculate subpopulations based on specific parameters, highlighting the objective reality of spermatozoa heterogeneity (Martínez-Pastor 2022). This complexity reflects molecular and genetic variability, including differences in DNA fragmentation and chromosomal alterations (Ogle *et al.* 2021). In essence, creating a universal solution for male infertility is difficult due to the heterogeneity and complex characteristics exhibited by spermatozoa. The absence of a ‘one drug fits all’ approach highlights the need for innovative and personalised strategies in addressing male fertility issues.

**Figure 1:**
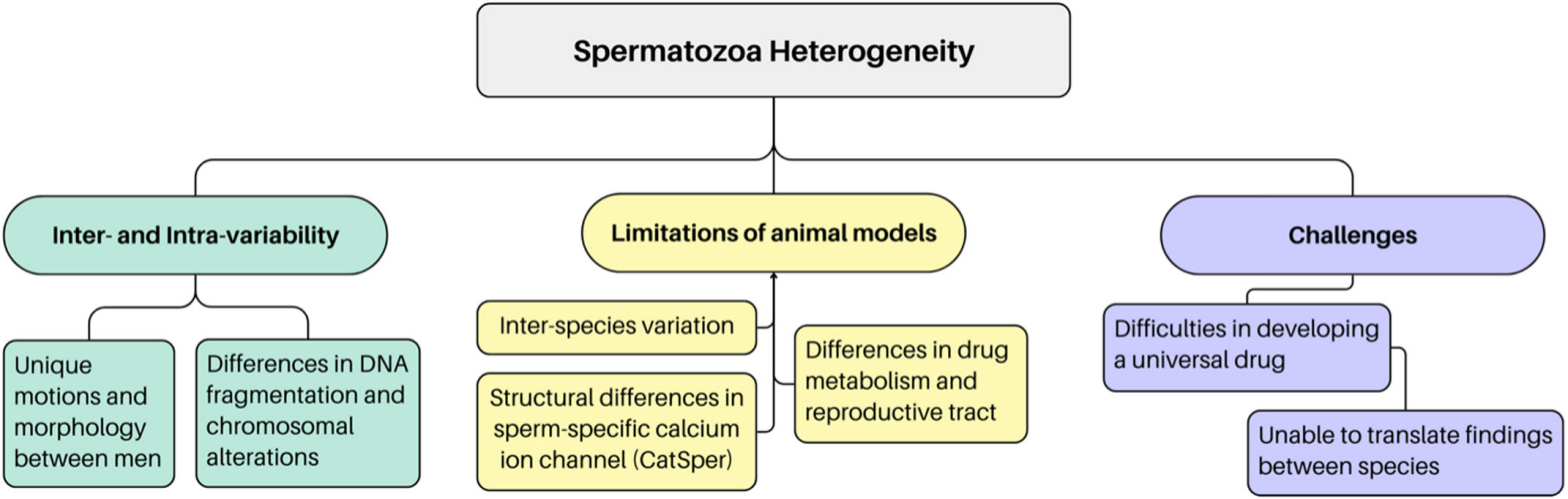
Variability in spermatozoa leads to additional challenges in drug development for male subfertility. Spermatozoa heterogeneity exists both between individuals and within the same individual over time, complicating the research process. Furthermore, the absence of appropriate animal models and generic challenges in drug development compound the difficulties inherent in the discovery process.

Furthermore, the limitation of animal models presents unique challenges due to the significant variations in spermatozoa morphology among different species. For example, the spermatozoa-specific calcium ion channel (CatSper), although critical to sperm function, exhibits notable structural (Zhao *et al.* 2022) and genetic (Lobley *et al.* 2003) differences between mice and humans. Moreover, extensive research has been conducted on spermatozoa morphology across diverse species, emphasising the evolutionary perspectives and adaptations in response to specific fertilisation environments. This means that translating findings to human applications faces constraints due to variations from other species. Factors such as differences in drug consumption, absorption, and the unique physiology of the genital tract contribute to the limitations in direct application to humans, adding complexity to drug development (Tautermann 2020). Therefore, unlike other fields of medical research, where animal testing serves as a reliable foundation for human applications, the intricate and species-specific adaptations in human spermatozoa make it difficult to translate findings across different species. A drug effective in one species may not yield the same results in humans due to these diverse adaptations. Consequently, researchers are restricted by these complex biological differences (Fig. 1) in developing targeted and effective therapies for male infertility.

### Idiopathic – the absence of an aetiology

One of the primary obstacles in developing a drug to address IMI is our limited grasp of basic spermatozoa physiology, as well as the molecular and cellular dynamics involved in fertilisation. IMI is likely due to a complex interplay between multiple causes, such as genetic, environmental, and hormonal factors (Cairo Consensus Workshop Group 2019). The absence of a defined aetiology and therefore a lack of known molecular targets or cellular receptors is a major limitation and significant hurdle for effective drug development. Consequently, the identification of potential drugs aimed at enhancing spermatozoa function and fertilisation is challenging.

### Drug development pathway

Drug development is a protracted process, characterised by scientific, regulatory, and economic challenges. The timeline runs from initial discovery to market approval, spanning years, if not decades (Martins da Silva *et al.* 2017, Tautermann 2020). The financial burden of translational research, coupled with extensive clinical trials and regulatory compliance, contributes to heightened attrition rates within the pharmaceutical industry, with only 1:5000 drugs successfully navigating the approval process from the laboratory to clinical use (Palanki & Bose 2023).

Navigating the regulatory landscape is a critical aspect of drug development, particularly in Reproductive Medicine. Adhering to stringent safety and efficacy standards requires meticulous documentation, comprehensive testing, and collaborative engagement (National Academies of Sciences, Engineering, and Medicine *et al.* 2021). Challenges in fertility drug research, including ethical concerns and potential transgenerational effects, add to the complexity, while managing collaborations across diverse global settings presents logistical challenges (Tautermann 2020, Kimmins *et al.* 2023).

Despite the global market for fertility-improving drugs exceeding USD 30 billion, infertility remains disproportionately overlooked in healthcare discussions (De Jonge & Barratt 2019). Furthermore, successful drug development necessitates understanding market niches and needs, anticipating competition, and adapting to healthcare policy changes (De Jonge & Barratt 2019, Tautermann 2020, Palanki & Bose 2023). Moreover, while conducting clinical trials is fundamental, challenges persist in recruitment, retention, and societal influences, especially in male fertility (Tautermann 2020, National Academies of Sciences, Engineering, and Medicine *et al.* 2021, Palanki & Bose 2023). A robust pharmacovigilance framework is also critical for continued post-market surveillance (National Academies of Sciences, Engineering, and Medicine *et al.* 2021).

The global decline in spermatozoa quality represents a looming threat to population health (Kimmins *et al.* 2023). Prioritising treatments for male infertility is not merely an option but a critical imperative. Neglecting male infertility could lead to a catastrophic decline in the human population as well as increasing budgetary pressure on an ageing society. It is long overdue for male infertility to be recognised and given a ‘seat at the high table’ alongside cancer and cardiovascular disease (CVD), as it undeniably merits a prominent position in our healthcare priorities.

### Crawling before running – the initial steps

While drug development techniques are available, as previously explored, there exists a pressing need for robust tests capable of analysing inherent spermatozoa function due to the limitations of traditional semen analysis. Such tests are essential to comprehend the complex nature of spermatozoa dysfunction and, in turn, allow a deeper understanding of how drugs affect spermatozoa, both in laboratory settings (*in vitro*) and within the body (*in vivo*).

Furthermore, in navigating the intricate landscape of male infertility research, collaboration emerges as a prerequisite for meaningful progress. Engaging in national collaborative trials, marked by shared resources and standardised measures, offers a transformative approach to overcoming challenges in prominence and resource allocation. By amalgamating expertise and resources from diverse regions, a more comprehensive understanding of male infertility can be achieved. At the core of this collaborative endeavour lies the need for a structured framework that sets a benchmark for excellence and cultivates a collective vision within the field. Multicentric projects and public–private partnerships hold the potential for broader impact. Transitioning from person-centric to project-centric funding aligns incentives, fostering a culture where collaboration becomes indispensable. This convergence of research practices on a global scale not only enhances the reliability of findings but also accelerates progress by minimising redundancies.

## Empirical therapy and unlicensed treatments

### Hormonal therapy

The efficacy of hormonal treatments to treat ovulatory disorders in females is well-established, prompting the hypothesis, perhaps not unreasonably, that similar treatments could be beneficial for disorders of spermatogenesis in males. Hormonal approaches include clomiphene citrate (Kumar *et al.* 2006, Huijben *et al.* 2023), letrozole (Yang *et al.* 2022), gonadotrophins (Esteves *et al.* 2023) and androgens (Kumar *et al.* 2006), summarised in Table 1.

**Table 1:** Empirical hormonal treatments for men with idiopathic male infertility.

Drug Class	Drug Name	Mechanism of Action		Challenges	
		Mechanism	Reference	Challenge	Reference
SERM	Clomiphene citrate	Activates oestrogen receptors and increases the secretion of gonadotrophins and the pulse amplitude of LH and FSH by partially inhibiting the negative feedback on the hypothalamus. The gonadotrophins act on the testes to increase testosterone production.		Paradoxical decrease in serum testosterone levels.	Helo *et al.* (2017)
		Two diasterioisomers Enclomiphene: oestrogen antagonist, shorter half-life (10.5 h), enhances testosterone and spermatogenesis. Zuclomiphene: oestrogen agonist, long half-life (30 days), dose accumulation impairing spermatogenesis.	Helo *et al.* (2017)	Paradoxical decline in semen parameters (% men) Decreased spermatozoa count (19%) Lower spermatozoa concentration (21%) Poorer spermatozoa motility (17%) Decrease in total motile spermatozoa count (24%)	Gundewar *et al.* (2021)
AI	Letrozole, Anastrozole	Block P450 aromatase activity, increasing endogenous testosterone levels. Increase in FSH and LH due to reduced negative feedback with lower serum oestradiol.	Yang *et al.* (2022)	Up to 17% men report loss of libido. More than 20% of men experienced fatigue and 15% reported hair loss. 10% men had liver dysfunction	Yang *et al.* (2022)
Gonadotrophins	FSH	Stimulation of Sertoli cells. Transfer of nutrients to developing germ cells. Promotes spermatogonia proliferation and maturation. Activates androgen receptors on Sertoli cells for spermatogenesis.	Esteves *et al.* (2023)	Poor cost-effectiveness	Santi *et al.* (2015)
				Low certainty of evidence.	Cannarella *et al.* (2020)
Androgens	Testosterone	Binds to androgen receptors on Sertoli cells. Promotes the proliferation and differentiation of spermatogenic cells, including spermatogonia, spermatocytes, and spermatids, and maturation during spermiogenesis to form spermatozoa.		Sustained azoospermia Hepatic dysfunction Gynecomastia Cholestasis	Kumar *et al.* (2006)

AI, aromatase inhibitor; FSH, follicle-stimulating hormone; LH, luteinizing hormone; SERM, selective oestrogen receptor modulator.

#### Selective estrogen receptor modulator

Off-label use of selective estrogen receptor modulator (SERM), specifically clomiphene citrate, has found some benefits in improving semen parameters (Kumar *et al.* 2006, Huijben *et al.* 2023). A recent meta-analysis explored the potential of clomiphene citrate to improve spermatozoa concentration and motility (Huijben *et al.* 2023). The analysis, while inclusive of diverse studies, lacked a specific focus on males with abnormal semen parameters. The wide-ranging semen analysis characteristics included those with normal values, creating complexity in the interpretation of the results. Importantly, the authors highlight significant study heterogeneity and the absence of high-quality randomised trials. These limitations raise concerns about the reliability of the findings in definitively establishing the efficacy and safety of clomiphene citrate in enhancing male fertility (Huijben *et al.* 2023). Despite showing promise, the lack of robust experimental designs and the heterogeneity among studies necessitate careful interpretation. And while generally reassuring, the safety and efficacy of clomiphene citrate in male infertility remain uncertain. Notably, a systematic review of 11 studies (384 men) reported a paradoxical decline in semen parameters following clomiphene citrate therapy. In men treated with clomiphene, 19% had a decrease in spermatozoa count, 21% had lower spermatozoa concentration, 17% had poorer spermatozoa motility, and 24% saw a decrease in total motile spermatozoa count. Crucially, the deterioration of semen parameters did not recover following the discontinuation of therapy in 1:6 (17%) individuals, so empirical clomiphene is apparently not risk-free (Gundewar *et al.* 2021).

#### Aromatase inhibitor

The first report of the use of aromatase inhibitors (AI) to treat male infertility involved a 31-year-old male with non-obstructive azoospermia and normal gonadotrophin levels (Patry *et al.* 2009). A testis biopsy after 4 months of treatment with letrozole showed normal spermatogenesis. Although used empirically, there is a lack of large-scale randomised controlled studies concerning the use of AIs to treat oligozoospermic or azoospermic men. AIs such as letrozole and anastrozole block the action of P450 aromatase and thus increase endogenous testosterone (T) levels. A secondary benefit of aromatase inhibition is a net increase in follicle-stimulating hormone (FSH) and luteinizing hormone (LH) due to reduced pituitary and hypothalamic negative feedback in the context of lower serum oestradiol (E2). Whilst this is likely to be a beneficial intervention for those with a deranged T:E2 ratio, whether this translates to improved male fertility for IMI is much less certain. Nonetheless, poor semen quality and attenuated sexual function secondary to obesity-related hypogonadism have been reported to improve with AI treatment (Yang *et al.* 2022). A variety of side effects are reported with AIs (Yang *et al.* 2022). Notably, up to 17% of men report loss of libido, which is a potentially significant issue for those trying to conceive naturally.

#### Gonadotrophins

Whilst the use of gonadotrophins (FSH and HCG/LH) is effective for true pituitary deficiency, their use has been widely debated in the management of IMI. FSH, a key regulator of spermatogenesis, consists of α and β subunits, the latter being FSH receptor (FSHR)-specific. Consequently, a deficiency in FSH adversely affects spermatozoa production (Esteves *et al.* 2023). Notably, a meta-analysis by Cannarella highlighted the potential of high-dose FSH in improving spermatozoa parameters. Despite promising findings, limitations such as small study numbers, high heterogeneity, and unaccounted co-factors like baseline health and lifestyle factors challenge the conclusive evidence regarding the substantial impact of FSH administration on male fertility. The lack of high-quality trials has led the authors to caution against overestimating the efficacy of FSH in improving idiopathic male infertility (Cannarella *et al.* 2020). A comprehensive evaluation through a Cochrane review has also shed light on this contentious topic. The analysis presented moderate-quality evidence that supports a positive influence on pregnancy rates. However, the authors found very low-quality evidence hinting at increased live birth rates (Attia *et al.* 2013). Santi observed that achieving a spontaneous pregnancy necessitates empirical FSH treatment in up to ten men, whereas initiating treatment in 18 men is essential for a successful pregnancy through ART (Santi *et al.* 2015). The hesitation in routinely providing FSH to men with IMI is therefore rooted in the lack of cost-effectiveness, particularly for patients self-funding their treatment in comparison to ART.

#### Androgens

Administration of androgens, traditionally considered essential for spermatogenesis, hinders rather than improves spermatogenesis in men due to suppression of endogenous testosterone. This unforeseen consequence highlights the complexity of hormonal interactions in male reproductive health, necessitating a re-examination of therapeutic approaches (Kumar *et al.* 2006).

As a result, despite the fact that various hormone approaches have been tried, the absence of comprehensive randomised controlled trials demonstrating the benefits of empirical hormonal therapy in increasing live birth rates has made it impossible to confidently recommend a medication for IMI.

### Phosphodiesterase inhibitors

Sperm motility, capacitation, and the acrosome reaction are regulated by signal transduction systems involving cyclic adenosine monophosphate (cAMP) and cyclic guanosine monophosphate (cGMP) as second messengers. Levels of cAMP and cGMP are controlled by two key enzymes, adenylyl cyclases, which catalyse their synthesis, and phosphodiesterases (PDEs), which are involved in their degradation. The intracellular availability of cAMP and cGMP therefore rests on the balance of synthesis versus degradation. Increased levels of intracellular cAMP result in an enhancement of spermatozoa motility and viability (Tardif *et al.* 2014).

PDEs are a family of related phosphohydrolases that selectively catalyse hydrolysis of the 3’ cyclic phosphate bonds of cAMP or cGMP, or both. There are multiple forms of PDEs with different kinetic and regulatory properties, classified into 11 different families and comprising 21 different gene products. Multiple proteins can be transcribed from these genes, due to alternative transcription start sites and splicing of precursor molecules (Beavo 1995). Any cell type can express multiple PDEs. The exact nature and localisation of the expressed PDEs largely specifies the regulation of intracellular cAMP and cGMP.

Methylxanthines belong to the first generation of PDE inhibitors and represent a group of compounds including, amongst others, theophylline, caffeine, and pentoxifylline (Sharpe *et al.* 2020). Publications over 40 years ago first identified positive effects on spermatozoa motility following *in vitro* exposure to the non-specific phosphodiesterase inhibitor (PDEI), pentoxifylline (De Turner *et al.* 1978). Pentoxifylline is thought to stimulate flagellar motility by increasing intracellular cAMP (Sharpe *et al.* 2020). However, only a very low concentration of pentoxifylline is detectable in seminal fluid after oral administration (Schramm & Hinze 1980), leading to speculation on its mode of action. One theory proposed that improved microcirculation within the epididymis and male accessory glands resulted in improved spermatozoa maturation (Schill 1982), another suggested that pentoxifylline may increase seminal fructose levels (Marrama *et al.* 1985).

A wealth of literature has been generated on the effects of non-specific PDEIs on spermatozoa, describing both positive and negative effects (Drobnis & Nangia 2017). Both theophylline and caffeine increase intracellular concentration of cAMP and the rate of progressive motility, fructose utilisation, and protein synthesis in ram spermatozoa (Salem *et al.* 1992). Theophylline has also been demonstrated to markedly increase the motility of epididymal-derived human spermatozoa. Similarly, caffeine increases spermatozoa motility and metabolism when added to semen (Drobnis & Nangia 2017). However, caffeine also promotes spontaneous acrosomal reaction, thus counteracting any benefit from its role as a motility stimulant. Increases in spontaneous acrosome reaction have been variably described in association with *in vitro* use of pentoxifylline (Tesarik *et al.* 1992). Importantly, pentoxifylline, used in the context of IVF, shows no difference in fertilisation rates compared with controls (Aizer *et al.* 2021). Cleavage characteristics, morphological quality of embryos, and pregnancy rates were also not significant between groups.

The disappointing lack of demonstrable difference in IVF fertilisation rate or on downstream embryo development made pentoxifylline obsolete as a spermatozoa preparation tool for IVF. Nonetheless, spermatozoa treatment with pentoxifylline prior to intrauterine insemination (IUI) for male factor subfertility was found to significantly increase pregnancy rates. In 61 cycles following standard procedure, the overall pregnancy rate was 11.5%, compared to 27.5% in 40 cycles with pentoxifylline treatment (*P* < 0.05). Perhaps not unsurprisingly, there were no significant differences in pregnancy rates in a further 49 cycles (10 standard IUI vs 39 pentoxiphylline treated spermatozoa) where the indication for fertility treatment was other than male factor (Negri *et al.* 1996). This was also confirmed by Stone et al, who found highest overall pregnancy rates following IUI where spermatozoa were pre-treated with pentoxifylline (Stone *et al.* 1999). However, historical improvement in IVF pregnancy rates, as well as the introduction of ICSI, left IUI under-utilised as a treatment modality (Tardif *et al.* 2014).

There is a strong argument that the improvement of spermatozoa motility is key to facilitating fertility. The existence of new generation and type-specific isoforms makes PDEs very attractive as drug targets and may yet serve as a potential adjunctive therapeutic option for improving male fertility (Tardif *et al.* 2014). Tardif found that PDEI, such as Papaverine and Tofisopam, could improve semen parameters, demonstrating sustained improvement in motility and kinematics. A list of compounds screened by Tardif (2014) can be found in Table 2.

**Table 2:** Compounds screened by Tardif et al (2014).

Dipyridamole	Mesopram
Ro 20-1724	Nicardipine
Cilostamide	Nimodipine
Vinpocetine	Cilostazol
Zardaverine	Rolipram
BRL 50481	Zaprinast
Pentoxifylline	IBMX
Papaverine	Milrinone
Theophylline	Tadalafil
Tofisopam	MMPX (8-MeO-lBMX)
Ibudilast	EHNA hydrochloride
Caffeine	RS 25344 hydrochloride
CP 80633	T 0156 hydrochloride
YM 976	W-7 hydrochloride
ICI 63197	W-9 hydrochloride
MY-5445	CGH 2466 dihydrochloride
Siguazodan	Etazolate hydrochloride
Bay-73-6691	Trequinsin hydrochloride
Bay-60-7550	Sildenafil citrate
(S)-(+)-Rolipram	A-7 hydrochloride
(R)-(−)-Rolipram	Anagrelide hydrochloride
Irsogladine maleate	

Adapted from Tardif *et al.* (2014) - Supplementary Table S1

Furthermore, trequinsin, a potent inhibitor of phosphodiesterase 3 (PDE3), has been reported for its positive impact on spermatozoa motility and function. Studies have demonstrated the efficacy of trequinsin in spermatozoa samples under capacitating conditions, showcasing its influence on hyperactivation and penetration into a viscous medium (McBrinn *et al.* 2019, Gruber *et al.* 2024). As a CatSper agonist, trequinsin exhibited the ability to modulate spermatozoa potassium channel activity, elevate cGMP (while not affecting cAMP), and induce calcium dynamics akin to progesterone. This unique combination of effects positions trequinsin as a versatile, multi-target compound capable of addressing specific molecular and functional impairments in spermatozoa function (McBrinn *et al.* 2019). Conversely, sildenafil, a specific inhibitor of PDE5 and a powerful therapy for male erectile dysfunction, enhances spermatozoa motility *in vitro*, but with an associated nearly doubling of the rate of spontaneous acrosome reaction, which clearly carries an adverse effect on male fertility (Glenn *et al.* 2007). Furthermore, there are significant concerns regarding the detrimental effects of sildenafil on fertilisation rates and embryo development, as shown in a mouse model (Glenn *et al.* 2009). Conversely, tadalafil significantly decreases spermatozoa progressive motility after oral administration, possibly due to a PDE11 inhibitory effect (Pomara *et al.* 2007). The use of PDE inhibitors clearly offers some very exciting concepts for future experimentation and discovery.

### Antioxidants, vitamin and dietary supplements

While the molecular basis of male infertility remains elusive, oxidative stress has emerged as a prominent player in poor spermatozoa function. The role of reactive oxygen species (ROS) is well recognised and vital for normal spermatozoa function, such as chromatin condensation and capacitation. However, an excess of ROS damages spermatozoa DNA and cell membrane integrity through oxidation and peroxidation reactions, with a spectrum of downstream effects on motility and the ability to fertilise an egg, cell death, as well as potential implications for pregnancy and miscarriage (Aitken & Drevet 2020). Sperm DNA fragmentation significantly affects both miscarriage rates and birth weight in ART. Li *et al.* (2024) discovered that elevated DNA fragmentation levels are positively correlated with higher miscarriage rates. Additionally, they observed a negative correlation between DNA fragmentation and birth weight, suggesting that increased DNA fragmentation is linked to lower birth weights (Li *et al.* 2024).

To address this, oral antioxidants are used widely in the management of male infertility, most often in the form of vitamin and dietary supplements (VDS) (Agarwal *et al.* 2019, Khaw *et al.* 2020). However, a Cochrane review encompassing 10,303 subfertile men found low-certainty and very low-certainty evidence that the use of antioxidants led to improved pregnancy or live birth rates (de Ligny *et al.* 2022). Notably, findings from males, antioxidants, and infertility (MOXI) and folic acid and zinc supplementation trial (FASZT) studies suggest that antioxidant therapy for male infertility is not risk-free (Table 3) (Schisterman *et al.* 2020, Steiner *et al.* 2020).

**Table 3:** Adverse events seen with empirical use of antioxidants for IMI.

Treatment	Effect on Spermatozoa and Pregnancy Outcomes	Adverse Effect	Study
Antioxidants (MOXI trial)	No substantial changes in semen parameters	Unexpected decrease in spermatozoa concentration	Steiner *et al.* (2020)
Folic acid and zinc (FAZST trial)	No significant differences in semen parameters	Potential decline in overall semen quality Increase in spermatozoa DNA damage Increase in preterm deliveries Gastrointestinal symptoms (abdominal pain, nausea, vomiting)	Schisterman *et al.* (2020)
Antioxidants (Cochrane review)	Insufficient evidence to show benefit on spermatozoa or fertility outcomes	Gastrointestinal symptoms	de Ligny *et al.* (2022)

The MOXI trial was a multicentre double-blinded randomised controlled trial where participants were either administered a blend of antioxidants or a placebo. The trial found no substantial changes in semen parameters, spermatozoa DNA fragmentation, or pregnancy outcomes. Perhaps most startling was the discovery that spermatozoa concentration had actually decreased following antioxidant administration (Steiner *et al.* 2020).

The FAZST trial examined the impact of folic acid and zinc supplementation on semen parameters and pregnancy outcomes. The double-blinded, placebo-controlled trial of 2370 men across various centres found no significant differences in semen parameters. More worrisome was the discovery of a potential decline in overall semen quality associated with the folic acid and zinc supplementation, contrary to the anticipated positive effects. Furthermore, the study unveiled an unexpected twist – an increase in spermatozoa DNA damage linked to the supplementation, a finding that defied the common belief that antioxidants could reduce such damage. Remarkably, there were no discernible differences in pregnancy outcomes; however, the authors observed a rise in preterm deliveries in the study group. Moreover, individuals in the treatment group reported a greater incidence of gastrointestinal symptoms, including abdominal pain, nausea, and vomiting (Schisterman *et al.* 2020).

In the context of previous research examining the impact of antioxidants on male infertility, a 2022 Cochrane review found very low certainty evidence from 12 small to medium-sized randomised controlled trials indicating that antioxidant supplementation in subfertile males may enhance live birth rates for couples seeking fertility treatment. Additionally, low-certainty evidence suggests a potential increase in clinical pregnancy rates. However, the authors have highlighted the inconclusive nature of current studies (de Ligny *et al.* 2022). de Ligny and colleagues have cited serious biases, inadequate reporting of pregnancy outcomes, substantial attrition rates, and imprecision stemming from small sample sizes as significant limitations. Aligning with the findings of the FAZST trial, the review revealed no increase in miscarriage rates but did report the possibility of antioxidant supplementation leading to gastrointestinal symptoms (de Ligny *et al.* 2022). The European Society of Human Reproduction and Embryology (ESHRE) add-ons working group consensus conclusion is that the existing body of evidence remains inadequate to support the use of antioxidant therapy to improve live birth rates. Consequently, the authors have advised that antioxidants should not be routinely incorporated as an adjuvant during fertility treatment protocols (ESHRE Add-ons working group *et al.* 2023).

## Technological advancements shaping the landscape of drug discovery

### High throughput phenotypic screening

A contemporary trend in drug discovery has been the widespread adoption of large-scale cell-based (phenotypic) screening. This approach involves cost and resource-efficient testing of libraries comprising thousands of compounds, encompassing both established medicines and novel molecules, to identify those that enhance or inhibit a specific cellular phenotype. This approach is particularly relevant in addressing drug discovery for IMI, where clear and identifiable drug targets are lacking. High-throughput phenotypic screening (HTPS) has proven to be a powerful approach for identifying innovative chemical compounds where the physiological and biochemical mechanisms governing spermatozoa motility and function are unknown or incompletely understood. It has enabled the identification of promising candidates both for male infertility (Martins da Silva *et al.* 2017, Gruber *et al.* 2022) as well as non-hormonal contraception (Johnston *et al.* 2022).

**Figure 2:**
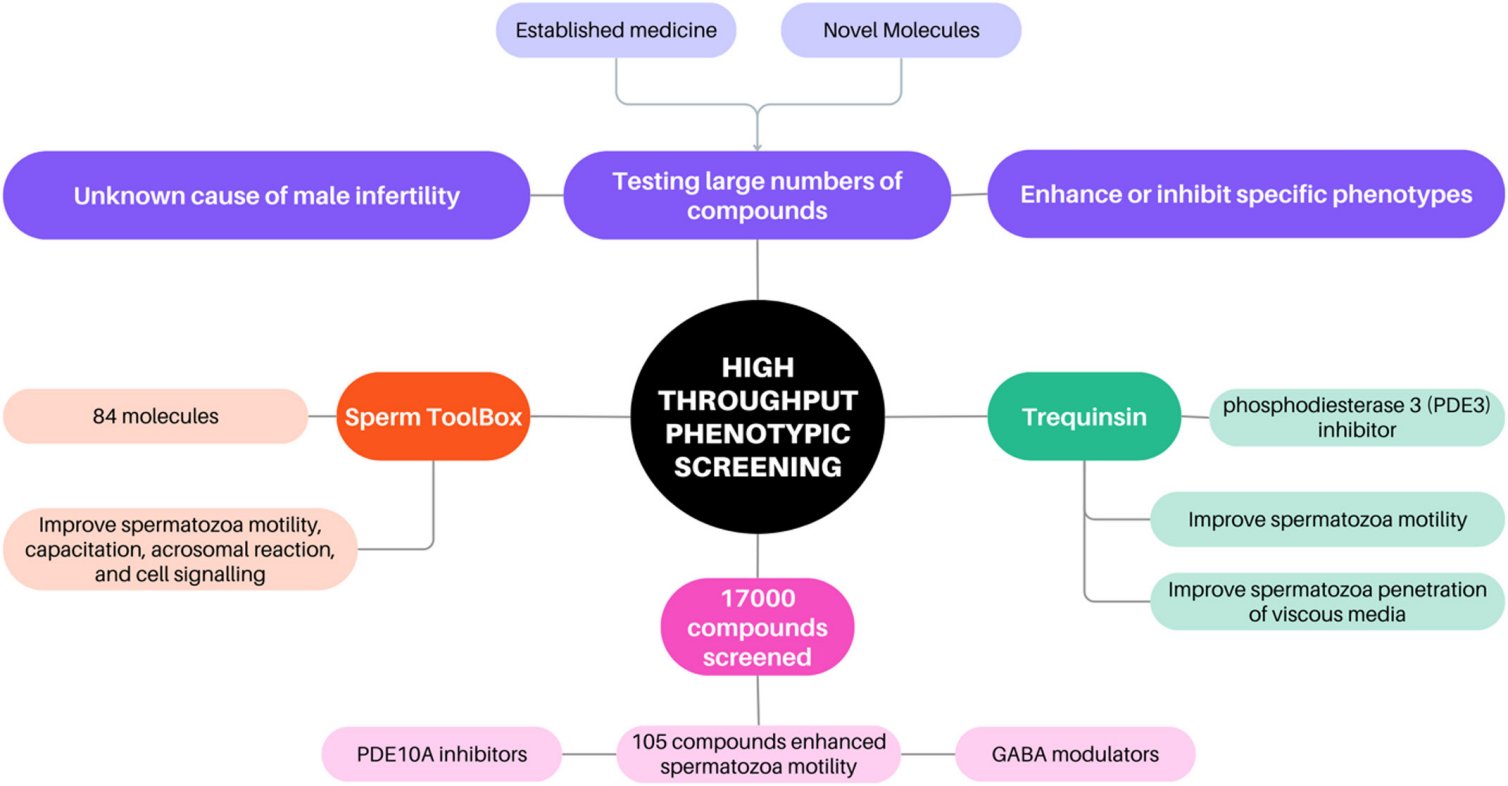
Application of high throughput phenotypic screening in drug development for male infertility. PDE, phosphodiesterase; GABA, gamma-aminobutyric acid.

Utilising HTPS (Fig. 2) on compounds aimed at enhancing spermatozoa motility, a total of 105 compounds exhibited a significant enhancement effect when compared to controls, including phosphodiesterase inhibitors (PDEI), specifically PDE10A inhibitors, as well as compounds associated with GABA signalling pathways (Gruber *et al.* 2022). Furthermore, Gruber has curated the Sperm Toolbox which consists of a collection of 84 meticulously chosen molecules with well-established effects on various aspects of spermatozoa function, including motility, capacitation, acrosomal reaction, and cell signalling to be used as a drug discovery tool going forward (Gruber *et al.* 2024).

While HTPS shows great promise in identifying compounds to enhance spermatozoa motility, certain limitations must be acknowledged. As the throughput of an assay intensifies, the complexity and resource requirements also rise. HTPS usually requires both specialised equipment including robotics and automation, as well as investment in a skilled team to develop and execute the workflow and interpret results and data (Moffat *et al.* 2017). Practical challenges of creating HTPS platform assays for IMI can also be significant, not least the considerable requirement for spermatozoa from volunteers, as well as reagent preparation and compound management (Vincent *et al.* 2022). HTPS also inherently carries the risk of identifying compounds with unknown or off-target effects, potentially leading to engagement in target-related activities or investigation into mechanisms of action associated with significant drug safety liabilities, for example, human ether-a-go-go-Related Gene (hERG) channel binding studies (Moffat *et al.* 2017, Vincent *et al.* 2022).

### Combinatorial chemistry

In the dynamic field of pharmaceutical research, the application of combinatorial chemistry emerges as a powerful tool, promising to reshape the landscape of drug discovery for male reproductive health. This innovative methodology involves the systematic synthesis and screening of diverse molecular combinations, generating expansive chemical libraries for meticulous high-throughput screening (Liu *et al.* 2017). The concept of dynamic combinatorial libraries introduces an adaptive facet to this methodology (Ramström & Lehn 2002). In the pursuit of solutions for male infertility, this involves generating libraries with evolving molecular compositions, responding to specific biological targets. This dynamism enhances adaptability, increasing the likelihood of pinpointing effective drug candidates that can optimise themselves based on intricate interactions with biological systems.

In the urgency of addressing male reproductive health issues, combinatorial chemistry provides a streamlined and efficient path toward ground-breaking medical solutions. The potential to discover drugs that target previously unexplored biological pathways is particularly promising. As the global focus intensifies on reproductive health, the significance of investing in and refining combinatorial chemistry for male infertility research becomes increasingly apparent.

Combinatorial chemistry in drug discovery for male infertility holds immense promise, propelled by its efficiency in accelerating the identification and optimisation of drug candidates. However, the journey is not without challenges, demanding a delicate balance between innovation and caution. As we navigate the intricate landscape of combinatorial chemistry, collaboration between disciplines and a steadfast commitment to ethical guidelines will ultimately determine the true impact of this revolutionary method on the future of male reproductive health interventions.

### High-throughput sequencing

High-throughput sequencing (HTS) is used to characterise biomarkers, including genes and proteins, derived from human tissue or blood. HTS techniques operate at different omics levels, including the genome, epigenome, transcriptome, proteome, and metabolome. Each level contributes to understanding disease development, progression, and treatment response (Yang *et al.* 2020).

The completion of the human genome project in 2015 marked significant progress in our understanding of genetic information (Green *et al.* 2015). Advances in transcriptomics, particularly at the molecular level, have greatly enhanced efficiency (Yang *et al.* 2020). These technologies enable the study of gene transcription levels, regulatory characteristics, and molecular mechanisms in disease processes and pathways affected by drug interventions. In the last decade, gene expression microarrays and RNA-seq have become routine methods for transcriptome profiling (Wajnberg & Passetti 2016). These technologies play a crucial role in drug discovery, shedding light on molecular mechanisms, composition, and potential therapeutic targets.

For example, RNA-Seq has proven effective in pinpointing insertions and deletions (INDELs) linked to various diseases, chemo-resistance, and unfavourable patient outcomes. The method boosts precision in identifying genetic variations and provides valuable insights into potential drug targets, offering a targeted approach to address specific genetic anomalies (Wajnberg & Passetti 2016). Digital RNA has also been developed, with perturbation of genes (DRUG-seq) platform utilising HTS. DRUG-seq is an innovative and cost-effective high-throughput RNA-Seq technique which has been specifically designed for drug discovery purposes. This method allows the comprehensive transcriptional profiling of thousands of genes simultaneously, covering hundreds to thousands of wells. Notably, DRUG-seq is adaptable to existing compound screening automations in industrial settings, streamlining the drug discovery process by efficiently analysing large gene datasets in a cost-effective manner (Ye *et al.* 2018).

High-throughput sequencing represents a cutting-edge technology in drug discovery, although the database of drugs developed through this method is not yet extensive. The dynamic nature of the drug discovery field ensures continuous research, and HTS holds a pivotal role within the omics approach, playing a crucial role in the systematic development of innovative pharmaceuticals (Ye *et al.* 2018).

### Drug repurposing

Drug repurposing is a process of identifying new therapeutic opportunities for existing drugs. It is a promising approach in drug discovery in terms of reducing the cost and time to bring new drugs to market. This concept covers identifying, developing, and commercialising new uses for drugs abandoned during pharmaceutical development (except those abandoned due to safety concerns) as well as modifying a drug formulation to allow a drug to enter a new market. Key examples include zidovudine, originally developed as an anti-cancer drug, which became the first anti-retroviral drug approved by the FDA for HIV/AIDS, sildenafil, originally developed for angina, but repurposed for erectile dysfunction; and minoxidil, an antihypertensive agent where retrospective analysis of trial data identified hair growth as an adverse event, now used as a topical preparation as the mainstay treatment for androgenetic alopecia.

#### Myeloperoxidase inhibitors

Myeloperoxidase (MPO) is a member of the superfamily of heme peroxidases, mainly expressed in neutrophils and monocytes. Elevated levels of MPO in the circulation are associated with inflammation and oxidative stress, and implicated in pathology including CVD, chronic kidney disease (CKD), myocardial ischaemia, stroke, and venous thrombosis (Correa *et al.* 2020). Given that oxidative stress is a common pathology encountered in male infertility, recent research has attempted to delve into the role of MPO in male infertility. MPO is produced by neutrophils in human semen. However, excessive MPO activity results in an overproduction of reactive oxygen species, compromising both semen quality and DNA integrity (Pullar *et al.* 2017). To address this issue, Campbell explored the potential of AZD5904, a myeloperoxidase inhibitor repurposed from AstraZeneca’s failed drug development, in alleviating oxidative stress caused by myeloperoxidase to enhance spermatozoa function. Their study unveiled a significant increase of more than 20% improvement during an *in vitro* spermatozoa penetration test (Campbell *et al.* 2021). This discovery presents a promising avenue for addressing male infertility and potentially enhancing fertility outcomes.

## Conclusions

Infertility has consequences that extend beyond reproduction. It causes significant psychological distress and financial hardship, as well as social consequences. Male infertility is also increasingly recognised to serve as a strong predictor of overall male health (Kimmins *et al.* 2023). There is an intricate web connecting male infertility to elevated mortality rates, diminished general health, and an increased susceptibility to CVD and cancer (Barratt *et al.* 2021, Kimmins *et al.* 2023). Given that men often hesitate to seek medical assistance (Kimmins *et al.* 2023), proactively screening for and addressing male infertility not only tackles reproductive challenges but also holds the potential for opportunistic intervention to mitigate risk and enhance overall health outcomes. New findings also shed some light on the potential familial effects of male infertility and ICSI, which link parental health to the well-being of their children (Rumbold *et al.* 2019). Effective drug interventions for male infertility and a reduced need for ICSI could attenuate potential transgenerational effects of ICSI still not fully described or understood.

There is a huge and unmet clinical need for drug discovery for male infertility. Addressing male infertility represents a strategy that extends beyond reproduction; it can mitigate gender inequalities and also includes the possibility of significant long-term health improvement. We absolutely need to focus resources and effort on tackling this global health problem.

## Declaration of interest

SCK is part of the Early Career Reviewing Panel for *Reproduction & Fertility,* and SMS is an Associate Editor for *Reproduction & Fertility*. SCK and SMS were not involved in the review or editorial process for this paper, on which they are listed as authors.

## Funding

This research did not receive any specific grant from any funding agency in the public, commercial, or not-for-profit sector. SMS holds grant funding from the Bill and Melinda Gates Foundationhttp://dx.doi.org/10.13039/100000865 and Innovate UKhttp://dx.doi.org/10.13039/501100006041, as well as researcher support from the Chief Scientist Officehttp://dx.doi.org/10.13039/501100000589 (CSO).

## Author contribution statement

SCK conceived and draughted the initial manuscript. SCK and SMS researched data for the article. All authors wrote and revised the manuscript critically. All authors approved the final version of the manuscript to be published.
